# Cervical Bone Graft *Candida albicans* Osteomyelitis: Management Strategies for an Uncommon Infection

**DOI:** 10.1155/2014/986393

**Published:** 2014-09-10

**Authors:** Carlo Brembilla, Luigi Andrea Lanterna, Andrea Risso, Giuseppe Bonaldi, Paolo Gritti, Bruno Resmini, Andrea Viscone

**Affiliations:** ^1^Department of Neurosurgery, Pope John XXIII Hospital, WHO Square No. 1, 24100 Bergamo, Italy; ^2^Department of Neuroradiology, Pope John XXIII Hospital, WHO Square No. 1, 24100 Bergamo, Italy; ^3^Department of Anaesthesia and Intensive Care, Pope John XXIII Hospital, WHO Square No. 1, 24100 Bergamo, Italy

## Abstract

*Candida* osteomyelitis in the current literature is an emerging infection. The factors contributing to its emergence include a growing population of immunosuppressed patients, invasive surgeries, broad-spectrum antibiotics, injection drug users, and alcohol abuse. The diagnosis requires a high degree of suspicion. The insidious progression of infection and the nonspecificity of laboratory and radiologic findings may contribute to a delay in diagnosis. The current case concerns a 27-year-old man with a spinal cord injury who, after undergoing anterior cervical fixation and fusion surgery, developed postoperative systemic bacterial infection and required long-term antibiotic therapy. After six months, a CT scan demonstrated an almost complete anterior dislocation of the implants caused by massive bone destruction and reabsorption in *Candida albicans* infection. The patient underwent a second intervention consisting firstly of a posterior approach with C4–C7 fixation and fusion, followed by a second anterior approach with a corpectomy of C5 and C6, a tricortical bone grafting from the iliac crest, and C4–C7 plating. The antifungal therapy with fluconazole was effective without surgical debridement of the bone graft, despite the fact that signs of the bone graft being infected were seen from the first cervical CT scans carried out after one month.

## 1. Introduction

*Candida* osteomyelitis is a relatively rare clinical entity [1, 2]. Although recent reports have significantly added to our understanding of the pathogenesis and epidemiology of* Candida* osteomyelitis, limited data exist regarding the appropriate management of this infection [3]. In fact, the Infectious Diseases Society of America (IDSA) guidelines for the management of* Candida* osteomyelitis are primarily based on case reports and case series [4]. The factors contributing to the emergence of this infection include a growing population of immunosuppressed patients (e.g., neutropenia, long-term steroid therapy), invasive surgeries, broad-spectrum antibiotics, the use of central venous catheters, injection drug users, and alcohol abuse [5].

*Candida* osteomyelitis can cause significant morbidity if not recognized early or effectively treated. The diagnosis requires a high degree of suspicion [6]. The insidious progression of infection and the nonspecificity of laboratory and radiologic findings may contribute to a delay in diagnosis [6, 7]. The vertebrae are the most commonly affected bones, followed by the sternum and ribs [6, 7]. In the current literature, a hundred cases of vertebral osteomyelitis due to* Candida* are reported [8–10]. Among these, there are only a few cases of vertebral* Candida* osteomyelitis following a spinal surgical procedure [8–10]. In the current literature,* Candida* osteomyelitis after anterior cervical spine surgery has never been reported.

## 2. Case Illustration

**Figure 1:**
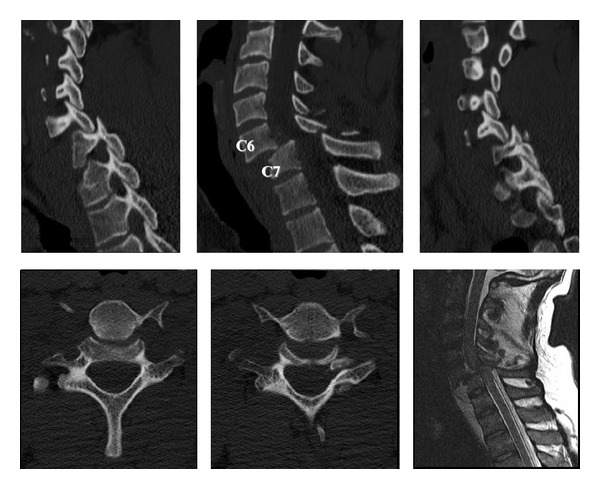
CT cervical scan showing a severe retrolisthesis of C6 on C5, with facet joint dislocation. MRI confirming the listhesis and demonstrating severe spinal cord injury with signs of myelopathy.

A 27-year-old man came to our attention due to a traumatic cervical injury during work, caused by a falling hayrick. On arrival, the neurological examination revealed paraplegia and diparesis, with deltoid and biceps muscle strength maintained bilaterally, and anaesthesia of the C6 dermatome. He had no medical history of infectious diseases or immunocompromise. He was afebrile. The complete blood count was within normal limits. Laboratory analysis revealed normal levels of creatinine, electrolytes, and serum proteins and included liver function tests. An initial computer tomography (CT) cervical scan revealed a severe retrolisthesis of C6 on C5, with facet joint dislocation, causing a considerable reduction in the cross section of the cervical spinal canal (Figure 1). A nuclear magnetic resonance imaging (MRI) of the cervical spine confirmed the listhesis and demonstrated severe spinal cord injury with initial signs of myelopathy.

**Figure 2:**
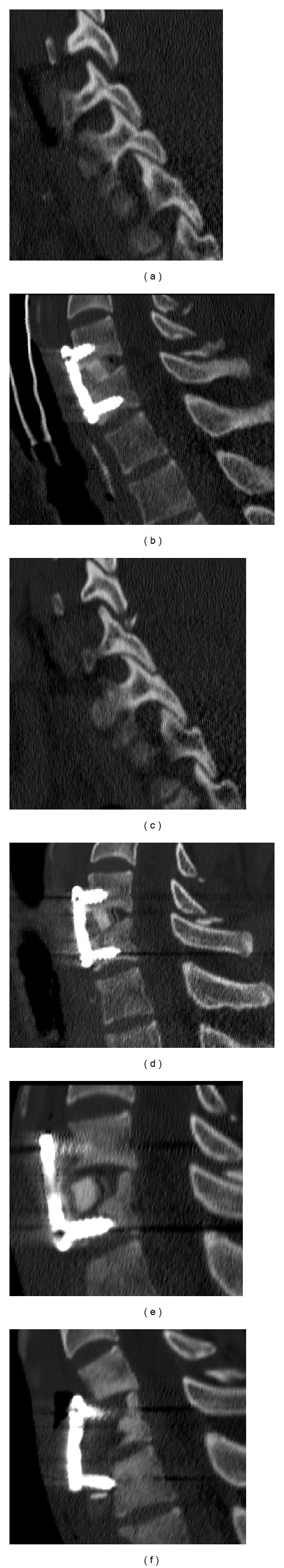
((a), (b), and (c)) Postoperative CT scan demonstrating a good reduction of the listhesis and of the luxation of the facet joints. (d) One month from CT scan demonstrating stability of the implant. (e) Three-month CT scan showing initial bone reabsorption around the cervical cage. (f) Six-month CT scan demonstrating an almost complete anterior dislocation of the implants caused by massive bone destruction and reabsorption.

The patient underwent an emergency neurosurgical intervention of close reduction and interbody anterior fixation-arthrodesis with a peek cage and anterior plating. A postoperative CT scan demonstrated a good reduction of the listhesis and of the luxation of the facet joints, with a satisfactory realignment of the vertebral bodies and correct positioning of the implants (Figures 2(a), 2(b), and 2(c)).

The postoperative period was characterized by neurological stability of the patient. Eight days after the surgical intervention he developed severe respiratory complications, which required a percutaneous tracheostomy. The patient had a central venous catheter. A few days later the patient developed a temperature, and secretions began to leak from the tracheostomy. The white blood cell count was 11,200 cells/mm³, C-reactive protein 17.5 mg/dL, and Procalcitonin 5.67 ng/mL. Samples for bacterial and fungal cultures from the secretion showed the presence of* Pseudomonas aeruginosa* and* Acinetobacter baumannii*; the blood cultures showed the presence of* Staphylococcus aureus* coagulase negative. The patient started systemic antibiotic therapy with ertapenem and teicoplanin. 50 days of antibiotic therapy led to a progressive improvement in clinical signs, laboratory findings, and negativization of the cultures.

The patient was moved to a rehabilitation center and maintained in a cervical brace. After 1 month from the surgical intervention the patient suffered no cervical pain and the neurological examination was unchanged. The cervical CT scan demonstrated stability of the implant (Figure 2(d)). After three months there was still no cervical pain; the patient was weaned from the use of the tracheostomy. The cervical CT scan demonstrated initial bone reabsorption around the cervical cage; the alignment of the vertebral bodies was still good (Figure 2(e)). The complete blood count, C-reactive protein, erythrocyte sedimentation rate, and Procalcitonin were normal. The blood cultures were negative. In view of the clinical stability and the absence of any infection being found, the authors considered the initial bone reabsorption to have been a mechanical problem. The patient continued to wear the cervical brace.

After six months, the patient still suffered no cervical pain and the neurological examination was unchanged. Just before being discharged from the rehabilitation center, a routine cervical CT scan demonstrated an almost complete anterior dislocation of the implants caused by massive bone destruction and reabsorption (Figure 2(f)). The alignment of the vertebral bodies was still good, but the cervical spine appeared unstable. The complete blood count was normal, while the C-reactive protein and erythrocyte sedimentation rate were slightly increased.

**Figure 3:**
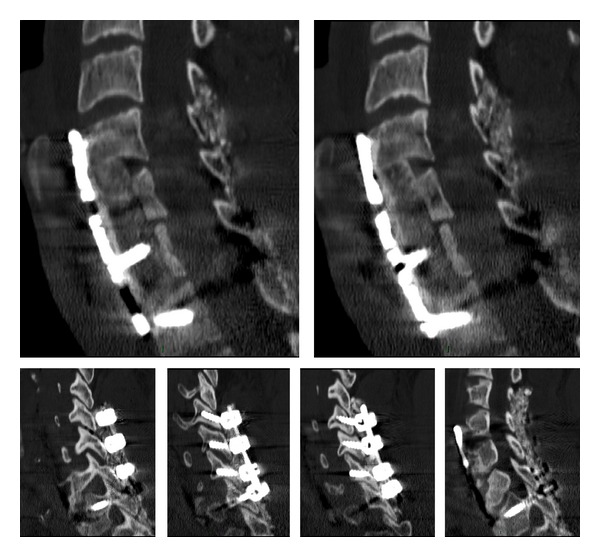
Postoperative CT scans showing posterior C4–C7 fixation and arthrodesis with lateral mass screwing, anterior C5-C6 corpectomy and tricortical bone grafting, and C4–C7 plating.

The patient underwent a neurosurgical intervention consisting of a first posterior approach with C4–C7 fixation and arthrodesis with lateral mass screwing followed by a second step consisting of an anterior corpectomy of C5 and C6, with a tricortical bone grafting from the iliac crest and C4–C7 plating (Figure 3). Intraoperatively, there was no infected or purulent tissue, but only a scar-like tissue that was sampled and sent for microbiological analysis. The culture results showed the presence of* Candida albicans*, susceptible to fluconazole. Based on antifungal susceptibility testing and according to the Infectious Diseases Society of America guidelines [4], treatment with fluconazole at a dosage of 400 mg (6 mg/Kg) daily was started.

**Figure 4:**
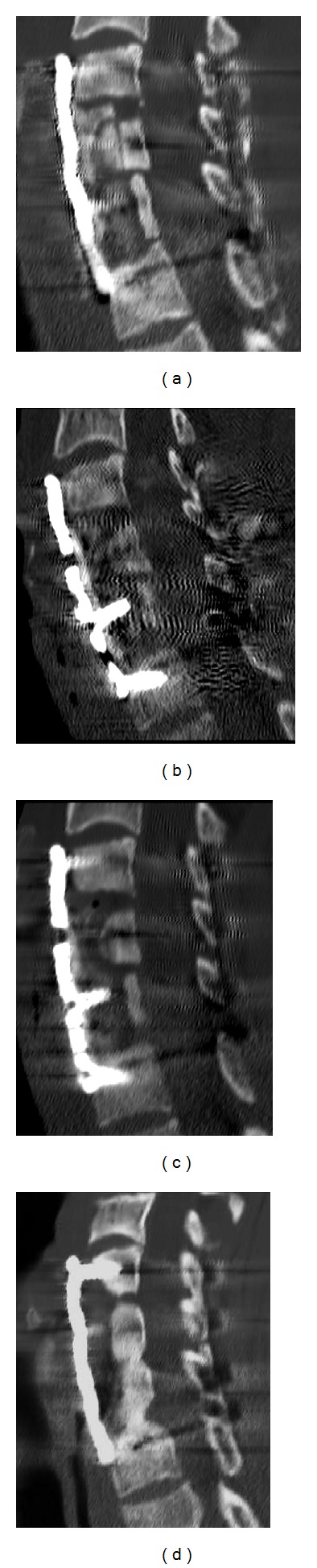
(a) One-month CT scans showing initial reabsorption of the tricortical bone graft with stability of the anterior plating. ((b) and (c)) Three- and 4-month CT scans showing extensive reabsorption of the bone graft with stability of the anterior plating. (d) One-year CT scan showing a fusion of the residual part of the bone graft and stability of the implant.

Cervical CT scans at one month showed initial reabsorption of the tricortical bone graft with stability of the anterior plating (Figure 4(a)). At 3 and 4 months, CT scans showed extensive reabsorption of the bone graft with stability of the anterior plating; the posterior implant seemed stable and the spinal segment was fused (Figures 4(b) and 4(c)). At 4 months C-reactive protein and the erythrocyte sedimentation rate were still slightly increased. The authors considered the possibility of a surgical debridement with removal of the anterior implant and allocation of a pedicle muscular flap (disengaging the distal head of the sternocleidomastoid muscle) on the infected site in order to bridge the antifungal therapy and sterilize the locus. This option would have required temporary immobilization with a halo jacket and a second surgical intervention. In view of the stability of the anterior and posterior implant and the presence of the posterior fusion, the authors decided to continue the antifungal therapy, monitoring the implant with CT and radiographic scans.

At 6 and 8 months, radiographic scans showed implant stability. At 8 months, the C-reactive protein and erythrocyte sedimentation rate were normalized. At one year, a CT scan showed a fusion of the residual part of the bone graft and anteroposterior-implant stability (Figure 4(d)). The C-reactive protein and erythrocyte sedimentation rate were normal. The antifungal therapy was stopped.

Now, after four years, the patient suffers no cervical pain and the neurological examination is unchanged. The white blood cell count, C-reactive protein, and erythrocyte sedimentation rate have always been normal, with no signs of the infection recurring. Up to now, the CT scans have shown anterior and posterior fusion and stability of the implants.

## 3. Discussion

Osteomyelitis is an inflammatory bone disease, frequently related to infection and characterized by progressive inflammation, bone destruction, and new bone growth [1, 2].* Candida* species are low virulence saprophytic microorganisms that inhabit the skin and mucous membrane of humans. Normally, bone is highly resistant to infection and* Candida* species are low virulence pathogens, which explains why* Candida* osteomyelitis rarely occurs [1, 2]. Although still uncommon,* Candida* osteomyelitis in the current literature is an emerging infection, with significant associated morbidity and mortality [3–6]. The factors contributing to the emergence of this infection include a growing population of immunosuppressed patients (e.g., neutropenia, long-term steroid therapy), invasive surgeries, broad-spectrum antibiotics, the use of central venous catheters, injection drug users, and alcohol abuse [3–11].

Osteomyelitis can occur in any bone including the tubular bones of the extremities and flat bones, but the vertebrae are the most commonly affected (64%), followed by the sternum and ribs (25%). The most affected vertebral level of infection is thoracic (43%) and lumbar (62%) and then cervical (8%) and sacral (7%). Some patients (13%) have shown evidence of multilevel spinal disease [8]. In the current literature, a hundred cases of vertebral osteomyelitis due to* Candida* are reported. Among these, only a few cases of vertebral* Candida* osteomyelitis have followed a spinal surgical procedure [8–10]. In the current literature,* Candida* osteomyelitis following anterior cervical spine surgery has never been reported.

Haematogenous seeding is the most common route of infection in associated osteomyelitis [11, 12]. The contiguous spread of infection to bone from adjacent soft tissues and joints or the direct inoculation of infection into the bone as a result of trauma or surgical procedures could represent further osteomyelitis mechanisms [13–15].* C. albicans* is the most common species involved in osteomyelitis. Less common species include* C. tropicalis*,* C. glabrata*, and* C. parapsilosis* [16, 17].

The diagnosis of* Candida* osteomyelitis requires a high degree of suspicion. The insidious progression of infection and the nonspecificity of laboratory and radiologic findings may contribute to a delay in diagnosis [6–14]. Long-standing back pain in the lower thoracic to lumbosacral spine is a typical symptom. Only a one-third deficit occurs in 20% of patients with candidal vertebral osteomyelitis [6–9]. Laboratory findings are nonspecific with an elevation of the erythrocyte sedimentation rate and C-reactive protein, and blood and urine cultures are rarely positive [12]. The nonspecific radiographic findings include disc space narrowing with irregular definition of the end plates and the gradual progressive destruction of the vertebral bodies on serial examinations [18]. Although one report described CT findings of a* Candida* infection showing end plate destruction as well as an epidural abscess, no features specific to* Candida* compared with other organisms have been noted [19]. Previous studies reported that MRI findings of* Candida* spondylitis are similar to those of pyogenic spondylitis. In immunocompromised patients, however, the absence of hypersignal intensity in a disc on a T2-weighted image may occur due to a lack of a cellular response [20]. The final diagnosis is determined by means of the culture of a biopsy specimen from the infected bone [12].

The Infectious Diseases Society of America (IDSA) revised guidelines for the management of* Candida* osteomyelitis are primarily based on case reports and case series [4]. According to the guidelines, treatment with fluconazole at a dosage of 400 mg (6 mg/Kg) daily for 6–12 months is recommended for the management of* Candida* osteomyelitis, with or without a 2-week “induction” phase with an echinocandin or an amphotericin B formulation. The azoles (such as fluconazole or voriconazole) are considered to have better bone penetration than amphotericin B products, which are considered poor in this regard [4]. Data on antifungal agent penetration into bone tissues are limited. Some authors have underlined the importance of surgical debridement of the affected area in conjunction with antifungal therapy for the treatment of* Candida* vertebral osteomyelitis, but this is not a commonly held view [4]. Given the lack of data, there is a need for more information regarding the selection of an appropriate antifungal agent, the duration of its administration, and the indications for therapeutic surgical intervention in the management of this disease.

The current case concerns a 27-year-old man with a spinal cord injury who developed postoperative bacterial infection requiring long-term antibiotic therapy. After six months, a CT scan demonstrated an almost complete anterior dislocation of the implants caused by massive bone destruction and reabsorption in* Candida albicans* infection. The patient underwent a second intervention consisting firstly of a posterior approach with C4–C7 fixation and fusion, followed by a second anterior approach with a corpectomy of C5 and C6, a tricortical bone grafting from the iliac crest, and C4–C7 plating. The antifungal therapy with fluconazole, administered according to the Infectious Diseases Society of America guidelines [4], was effective without surgical debridement of the bone graft, despite the fact that signs of the bone graft being infected were seen from the first cervical CT scans carried out after one month.
